# Echoes of precision: feedback, governance and ultrasound in prehospital care

**DOI:** 10.1186/s13049-025-01362-x

**Published:** 2025-03-21

**Authors:** Marco Tartaglione, Luca Carenzo

**Affiliations:** 1 Intensive Care and Prehospital Emergency Medical Service of AUSL Bologna, Ospedale Maggiore, Bologna, Italy; 2 Department of Anesthesia and Intensive Care Medicine, IRCCS Humanitas Research Hospital, Rozzano, Milano, Italy

## Introduction

The role of prehospital point-of-care ultrasound (PoCUS) in managing critically ill patients has expanded significantly. In this issue of the journal Naeem et al. [1] demonstrated the feasibility and safety of integrating PoCUS within a mature helicopter emergency medical services (HEMS) through continuous feedback and a robust governance structure. The paper suggests that if safely introduced, effective clinical reasoning can benefit from diagnostic tools that answer specific, clinically relevant questions, optimizing patient care and decision-making in a restricted time frame.

### The role of prehospital pocus

Like any other tool available to Emergency Medical Service (EMS) crews, the use of FAST (Focused Assessment with Sonography for Trauma), a rapid ultrasound protocol used to detect free fluid in the peritoneal, pericardial, and pleural cavities, provides an advantage in patient management when its opportunity, feasibility, and timing are properly understood within the system. However, indiscriminate use of FAST without a precise clinical query (FAST rather than PoCUS) may lead to fixation errors, diverting attention from other critical issues affecting the patient at that moment. A recent study by van der Geest et al. [2] suggested that prehospital ultrasound can overwhelm some practitioners, reducing their situational awareness and leading to missed clinical deteriorations. Their findings highlight the need for training and structured governance to mitigate cognitive overload and ensure that ultrasound use complements, rather than detracts from, comprehensive patient assessment.

Prehospital ultrasound, particularly the Pump, Pleura, and Pouring Blood (PPPB) protocol utilized by Naeem et al. [1], serves this scope aiming to rapidly identify life-threatening conditions. Thus, the reported sensitivity (56%) and specificity (90%) highlights the necessity for contextual interpretation of findings.

The clinical impact of prehospital ultrasound is well-documented, demonstrating improvements in patient management, pathway selection, and time-to-treatment [3]. Given its proven safety and positive impact on critical patient pathways, its broader implementation is a logical step forward.

Currently, in many European EMS systems, the use of prehospital ultrasound is largely contingent on the presence of a physician on board, a resource that is not always available. Moreover, the skill levels among physicians can vary significantly, leading to inconsistencies in its application. Relying solely on physician-staffed advanced units for the effective delivery of prehospital ultrasound is likely neither practical nor resource-efficient.

### Clinical governance in prehospital pocus

Clinical governance is a structured framework ensuring the safe, effective, and accountable delivery of medical care through standardized training, continuous performance, feedback, quality assurance, and error mitigation. The continuous feedback model employed by Naeem et al. [1] aligns with best practices, promoting ongoing skill development and quality assurance. However, as emphasized by previous works on the topic, governance frameworks must also facilitate error identification, continuous education, and adherence to standardized protocols [4].

Carenzo et al. [5] outline a comprehensive clinical governance model in intensive care medicine, which could be adapted for prehospital PoCUS. This model is structured around key pillars such as systems awareness, teamwork, communication, and leadership, which are essential for fostering a sustainable culture of quality improvement. The implementation of governance strategies should include clinical audits, real-time feedback loops, structured risk management, and standardized training programs to ensure high levels of diagnostic accuracy and consistency.

Expanding PoCUS beyond physician-only models, particularly in resource-constrained settings, necessitates scalable governance strategies. Remote supervision and tele-ultrasound have shown promise in supporting non-physician operators, enhancing diagnostic accuracy and decision-making [6, 7]. Establishing governance frameworks that integrate tele-ultrasound as a supervision tool could bridge the competency gap and ensure real-time oversight.

To enable the effective adoption of PoCUS at a system-wide level, governance structures should ensure:

1. The professional growth of all the members of the multiprofessional EMS family and the overall system.
2. A built-in safety net that continuously identifies and corrects deviations from standard of care.
3. Exposure of less experienced operators to expert perspectives, fostering continuous learning.
4. Consistently high-quality patient care, even in a system with high turnover and diverse professional backgrounds.

### **Training**, competency, and system efficiency

A structured training program, continuous performance feedback, and a robust review process are crucial for ensuring consistent PoCUS performance. Naeem et al.’s approach demonstrates that, with appropriate oversight, non-physician personnel can effectively perform PoCUS, broadening the reach of advanced diagnostics in prehospital care. However, balancing the benefits of prehospital PoCUS with potential impacts on scene time and resource allocation remains essential. Clinical governance should include protocols that prioritize rapid decision-making without compromising diagnostic accuracy.

Moreover, standardized certification pathways for prehospital PoCUS operators could enhance skill uniformity and reliability. Future governance frameworks should incorporate simulation-based training, real-time competency assessments, and structured credentialing to ensure a high level of diagnostic precision.

## Conclusion

The integration of prehospital PoCUS requires more than technical implementation: it demands a comprehensive governance framework that ensures quality, consistency, and clinician accountability. By embedding ultrasound use within structured clinical governance, EMS systems can enhance diagnostic precision, streamline patient pathways, and ultimately improve patient outcomes.

This remarkable example of clinical governance applied to prehospital PoCUS provides a structured framework for enhancing system efficiency and efficacy, serving as a guiding model for implementing meaningful improvements in practice. Future research should explore the long-term impact of governance models on diagnostic accuracy, skill retention, and patient-centered outcomes in prehospital care.

## Data Availability

No datasets were generated or analysed during the current study.
